# Phosphorylation systems in symbiotic nitrogen-fixing bacteria and their role in bacterial adaptation to various environmental stresses

**DOI:** 10.7717/peerj.8466

**Published:** 2020-02-11

**Authors:** Paulina Lipa, Monika Janczarek

**Affiliations:** Department of Genetics and Microbiology, Institute of Biological Sciences, Maria Curie-Sklodowska University Lublin, Lublin, Poland

**Keywords:** Symbiotic bacteria nitrogen fixation rhizobia adaptation stress conditions phosphorylation, Nitrogen fixation, Rhizobia, Adaptation to stress, Symbiosis, Protein phosphorylation, Soil environment, Two-component systems, Serine/threonine kinases and phosphatases, Phosphenolopyruvate-dependent phosphotranspherase systems

## Abstract

Symbiotic bacteria, commonly called rhizobia, lead a saprophytic lifestyle in the soil and form nitrogen-fixing nodules on legume roots. During their lifecycle, rhizobia have to adapt to different conditions prevailing in the soils and within host plants. To survive under these conditions, rhizobia fine-tune the regulatory machinery to respond rapidly and adequately to environmental changes. Symbiotic bacteria play an essential role in the soil environment from both ecological and economical point of view, since these bacteria provide Fabaceae plants (legumes) with large amounts of accessible nitrogen as a result of symbiotic interactions (i.e., rhizobia present within the nodule reduce atmospheric dinitrogen (N_2_) to ammonia, which can be utilized by plants). Because of its restricted availability in the soil, nitrogen is one of the most limiting factors for plant growth. In spite of its high content in the atmosphere, plants are not able to assimilate it directly in the N_2_ form. During symbiosis, rhizobia infect host root and trigger the development of specific plant organ, the nodule. The aim of root nodule formation is to ensure a microaerobic environment, which is essential for proper activity of nitrogenase, i.e., a key enzyme facilitating N_2_ fixation. To adapt to various lifestyles and environmental stresses, rhizobia have developed several regulatory mechanisms, e.g., reversible phosphorylation. This key mechanism regulates many processes in both prokaryotic and eukaryotic cells. In microorganisms, signal transduction includes two-component systems (TCSs), which involve membrane sensor histidine kinases (HKs) and cognate DNA-binding response regulators (RRs). Furthermore, regulatory mechanisms based on phosphoenolopyruvate-dependent phosphotranspherase systems (PTSs), as well as alternative regulatory pathways controlled by Hanks-type serine/threonine kinases (STKs) and serine/threonine phosphatases (STPs) play an important role in regulation of many cellular processes in both free-living bacteria and during symbiosis with the host plant (e.g., growth and cell division, envelope biogenesis, biofilm formation, response to stress conditions, and regulation of metabolism). In this review, we summarize the current knowledge of phosphorylation systems in symbiotic nitrogen-fixing bacteria, and their role in the physiology of rhizobial cells and adaptation to various environmental conditions.

## Introduction

The soil is an environment, which hosts an extremely diverse community of organisms, with high numbers of microorganisms (from 10^4^ to 10^9^ cells per gram of soil) (Poole, Ramachandran & Terpolilli, 2018). The largest microbial variety is observed in the rhizosphere, which is the soil surrounding the plant root. This zone is approximately 1-mm wide, and is the most intense area of biological and chemical activity, and thus the most dynamically changing niche in the soil (Kannan, Sithara & Chandru, 2015). Numerous compounds secreted by plant root (referred to as rhizodeposits), such as water-soluble ions, low-molecular weight compounds, carbohydrates, amino acids, organic acids, and other metabolic products cause biochemical changes in the soil (Haldar & Sengupta, 2015). Plant root secretome and abiotic factors, such as climate, low and high temperatures, humidity, pH, and light, influence the soil microbiome, affecting its biodiversity and adaptability (e.g., *Acidobacter* bacteria predominate in the soil under acidic conditions, while *Proteobacteria, Acinobacteria*, and *Firmicutes* prevail at neutral and alkaline pH). Consequently, soil microorganisms have developed a variety of metabolic strategies, including photosynthetic abilities, ammonia oxidation, and atmospheric dinitrogen (N_2_) fixation. Among them, biological fixation of N_2_ is extremely important not only for its ecological aspect, but also from an economical point of view. Globally, approximately 200 million tons of N per year are introduced into the environment via microbial fixation, an amount that is similar to that introduced by artificial N fertilizers (Roca et al., 2013; Rascio & La Rocca, 2013). Further, 70% of biologically fixed N_2_ comes from symbiotic systems, whereas only 30% comes from non-symbiotic N_2_ fixation. The non-symbiotic relationships involve heterotrophic bacteria living freely in the soil environment, such as *Azotobacter* spp., *Bacillus* spp., *Clostridium* spp., and *Klebsiella* spp., whose N_2_-fixing capacity varies from 10 to 20 kg N per hectare per year (Kumar et al., 2015). Another example of N_2_ fixation on a similar scale as that mentioned above is the associative N_2_ fixation conducted by microorganisms from *Azospirillum* spp. These bacteria establish associations with several types of grasses and cereals, such as wheat, oat, barley, rice, and maize (Fukami, Cerezini & Hungria, 2018). However, it was recently determined that free-living fixation represents the dominant biological source of N_2_ in many ecosystems, which lack of large numbers of symbiotic N_2_-fixers (e.g., tropical evergreen forests, moist tundra and alpine tundra, and temperate grasslands) (Reed et al., 2010; Reed, Cleveland & Townsend, 2011). Among microorganisms capable of fixing atmospheric N_2_, bacteria establishing symbiotic interactions with leguminous plants (Fabaceae) (e.g., pea, bean, soybean, clover, and alfalfa) play an important role (Williams et al., 2008). These N_2_-fixing symbiotic bacteria, collectively called rhizobia, belong to the large and diverse family *Rhizobiaceae* (order Rhizobiales, classes *α*- and *β*-Proteobacteria), which encompasses several genera (e.g., *Rhizobium, Sinorhizobium, Azorhizobium, Allorhizobium, Methylobacterium, Carbophilus,* and *Ciceribacter* (*α*-rhizobia); and *Burkholderia* and *Cupriavidus* (*β*-rhizobia)). Furthermore, bacteria from two other families, *Bradyrhizobiaceae* (*Bradyrhizobium* spp.) and *Phyllobacteriaceae* (*Mesorhizobium* spp*.*), possess N_2_-fixing symbiotic ability (Dresler-Nurmi et al., 2009; Weir, 2016). Currently, rhizobia are N_2_-fixing microorganisms that are the most studied and best described on a molecular level. These bacteria can live as free-living soil microorganisms and engage in symbiotic interactions with compatible host plants (Williams et al., 2008). Rhizobia infect legume root and induce formation of special organs called nodules, inside which they differentiate to bacteroids able to fix atmospheric N_2_. Thus, these bacteria provide nitrogen forms that the host plant can assimilate and make the host independent of the external input of this nutrient. For this reason, rhizobia play a significant role in the environment from both an economic and ecological point of view.

Changes in the soil conditions, occurrence of competition in this ecological niche, and various lifestyles of symbiotic bacteria require coordination of their cellular functions in response to signals of both extracellular and intracellular origin. To adapt to these different conditions, rhizobia have developed various strategies. These include numerous post-translational modifications of proteins (PTMs), which are involved in several signal transduction pathways (Kobir et al., 2011). PTMs affect a number of important protein features, such as their structure, activity, surface charge, and stability. They also influence their interactions with other molecules or subcellular location (Mijakovic, Grangeasse & Turgay, 2016). By contrast with eukaryotic organisms, only a few types of PTMs have been discovered in prokaryotes (e.g., glycosylation, methylation, phosphorylation, and acetylation) that are involved in signal transduction and pathogenesis, and may directly or indirectly change or abolish the interaction between proteins and other cellular components. Among these PTMs, phosphorylation is the most frequent and best-characterized modification (Kennelly, 2001; Kobir et al., 2011; Mijakovic, Grangeasse & Turgay, 2016). The phosphate group covalently attached to proteins by bacterial kinases is extremely reactive, which determines its biophysical properties, causing structural perturbation and changes in protein functionality. Many amino acids in a protein can act as potential acceptors of the phosphate group [including histidine (His), tyrosine (Tyr), serine (Ser), threonine (Thr), and aspartic acid (Asp)] (Johnson & Lewis, 2001). Based on the type of chemical bond formed with the phosphate group, amino acids can be divided into several groups. The first type of bond is a simple phosphoamide bond, when the phosphate group is attached to the hydroxyl residue of an amino acid (Ser, Thr, and Tyr); this type of bond is extremely stable chemically, and is resistant to both acids and hydroxylamine. The second type includes phosphorylation of basic amino acids [Arg, His, and lysine (Lys)] involving the formation of phosphoamidic bond, which is resistant to the action of base but is hydrolysable by acid and hydroxylamine. The third group encompasses phosphate modification of acidic amino acids [i.e., Asp and glutamic (Glu) acids], with acyl phosphate bond formation. The modifications of these amino acids are relatively unstable. The literature data also indicate the phosphorylation of protein cysteine (Cys) residues, with the formation of a phosphate phosphothiol bond (Cozzone, 1998; Mijakovic, Grangeasse & Turgay, 2016). Another important feature of phosphorylation is its reversibility (i.e., dephosphorylation), catalyzed by bacterial phosphatases. The phosphorylation/dephosphorylation cycle ensures precise regulation of many metabolic pathways in microorganisms. Differences in the stability of chemical bonds involving the phosphate group determine the ability to transfer phosphate group to the target proteins, leading to changes in their biological functions (Mijakovic, Grangeasse & Turgay, 2016). Based on recent data, three main phosphorylation mechanisms are present in prokaryotic organisms: two-component signal transduction systems (TCSs), phosphoenolopyruvate (PEP)-dependent phosphotransferase systems (PTSs), and phosphorylation of Ser, Thr, or Tyr residues in proteins (Kobir et al., 2011). Moreover, Arg-kinases phosphorylating proteins on this amino acid are known in bacteria (Mijakovic, Grangeasse & Turgay, 2016). To date, protein phosphorylation in prokaryotic organisms has been extensively described mainly in pathogenic bacteria. Until now, phosphorylation systems in symbiotic N_2_-fixing bacteria and their role in the adaptation of these microorganisms to various environmental conditions have not been an object of review reports. Therefore, in the present review, we summarize the current knowledge of phosphorylation systems in symbiotic bacteria and their role in rhizobial adaptation to various environmental conditions.

Experimental studies in rhizobia indicate that phosphorylation plays an essential role in many physiological processes in these bacteria and their adaptation to various environmental factors (e.g., exopolysaccharide and flagellum production, dicarboxylate transport, catabolite repression, phosphate utilization, N_2_ fixation, and adaptation to pH stress and microaerobic conditions) (Liu, Tian & Chen, 2015). We here present a concise overview of phosphorylation mechanisms in rhizobia and outline the current knowledge of the role of this PTM of proteins in cell signaling, coordination of vital functions, and bacterial adaptation to environmental stress conditions.

## Survey Methodology

In this review, we discussed the current literature data related to phosphorylation systems in rhizobia, and their functions in physiology and adaptation of these bacteria to various environmental conditions. References mentioned in the review were retrieved from PubMed up to September 2019. We used search terms such as phosphorylation in symbiotic bacteria, TCS system, PTS system, Hanks-type Ser/Thr kinases, Ser/Thr phosphatases, rhizobia, and nitrogen-fixing symbiosis. The considered references provide information about phosphorylation mechanisms in rhizobia, and their role in free-living bacteria and during symbiosis with host plant. Grouping and classification of rhizobial response regulators (RRs) were done based on conserved domains, according to papers of Galperin (2006) and Galperin & Nikolskaya (2007). The RRs encoded by genomes of rhizobial species have been counted and classified into individual families according to a type of their effector domain (DNA-binding, RNA-binding, enzymatic, and other). Protein sequences of ExoP and PssZ homologs were obtained from the NCBI public database. Alignment of protein rhizobial ExoP and PssZ homologs was performed using EMBL-EBI, 2019 available online (https://www.ebi.ac.uk/Tools/msa/clustalo */).* To obtain theoretical models of three-dimensional structures of ExoP and PssZ homologs, sequences of these proteins in FASTA format obtained from the NCBI database (http://www.ncbi.nlm.nih.gov/ *)* and a protein structure prediction server RaptorX (http://raptorx.uchicago.edu/ *)* were used. Several properties of ExoP and PssZ proteins were determined using Protein Molecular Weight Calculator (http://www.sciencegateway.org/tools/proteinmw.htm */)*, Isoelectric Point Calculator (http://isoelectric.org/calculate.php */)*, and program for secondary structure prediction of proteins (http://www.cbs.dtu.dk/services/NetSurfP/). Sequence identity and similarity were determined using BLAST program (https://blast.ncbi.nlm.nih.gov/Blast/).

## Two-component Signal Transduction Systems (TCSs)

TCSs are ubiquitous among bacteria. They occur exclusively in prokaryotes and archaea (Wang, 2012). They allow the microorganism to adapt to various environmental conditions, such as changes in nutrient availability, soil pH, temperature, redox status, osmolality, bacterial population density, and the presence of antibiotics and repellent plant metabolites. The number of TCS systems present in individual bacteria is closely correlated not only with the size of the genome, but also with the ecological niche these bacteria occupy (Galperin, 2006; Gao, Mack & Stock, 2007). Typically, TCS-encoding genes account for approximately 1–2% of the microbial genome, although it depends on many factors. Among such factors, the most important is the frequency of environmental changes in the ecological niche and microbial lifestyle (e.g., pathogenic bacteria possess up to 200 TCSs, whereas endosymbionts have fewer TCSs) (Galperin, 2006; Gao, Mack & Stock, 2007). For an example, QseBC and QseEF TCSs in enteric foodborne pathogens, such as enterohemorrhagic *Escherichia coli* (EHEC) and *Salmonella enterica* serovar Typhimurium, are involved in modulation of the expression of virulence genes in response to quorum sensing signals (i.e., autoinducer-3, epinephrine, and norepinephrine) from the microbiota or the host (Lustri, Sperandio & Moreira, 2017). Another TCS, KdpDE is involved in potassium homeostasis and intracellular survival of pathogenic bacteria, including *Staphylococcus aureus*, EHEC, *S. typhimurium*, and *Yersinia pestis* (Freeman, Dorus & Waterfield, 2013).

**Figure 1:**
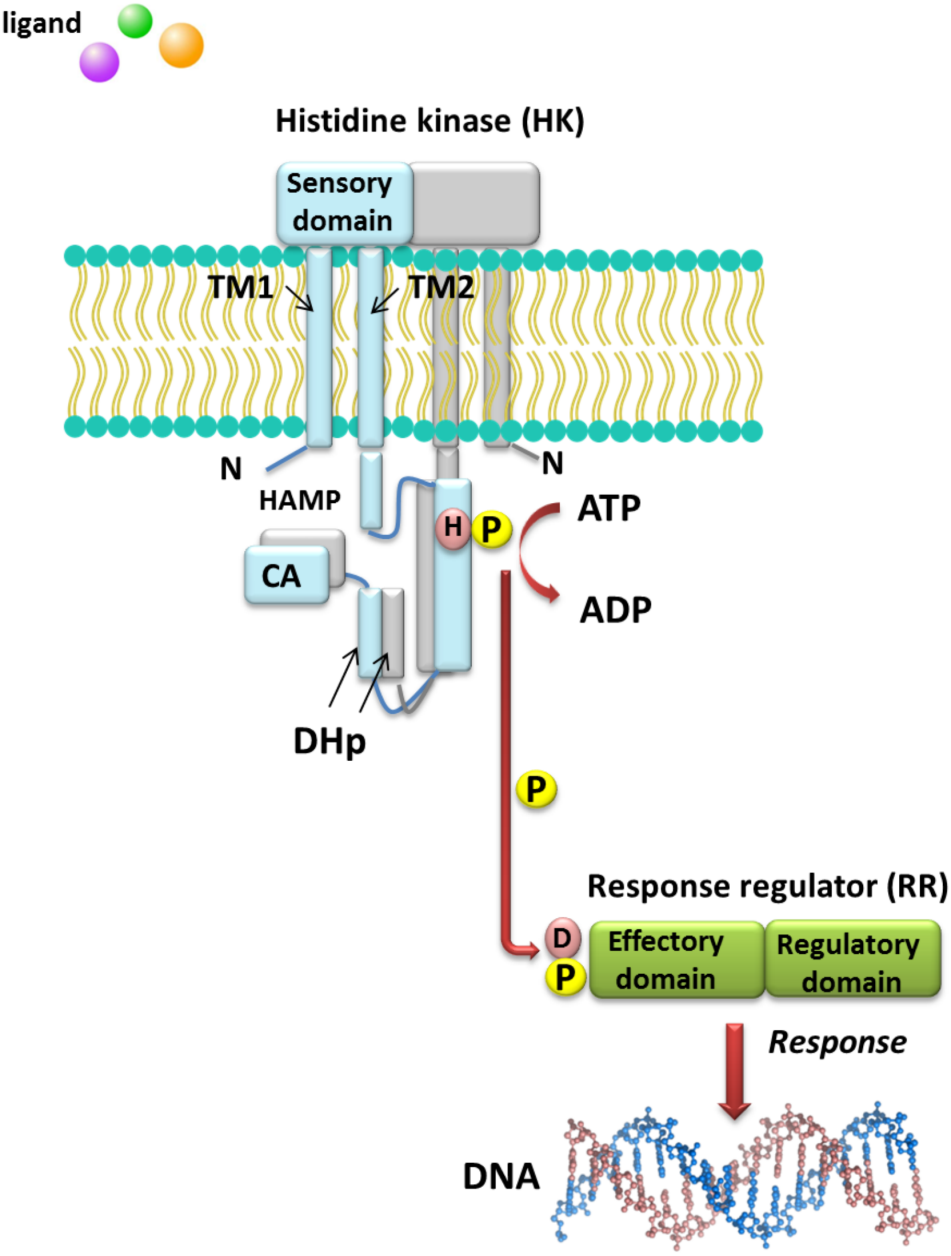
Model of signal transduction via the bacterial TCS system (based on data included in a study by Wang, 2012). TM, transmembrane domain; HAMP domain, name after the first letters of enzymes in which it occurs, i.e., histidine kinases, adenylate cyclases, methyl-accepting proteins, and phosphatases; CA, catalytic and ATP-binding domain; DHp, Dimerization Histidine phosphotransfer domain; H, histidine; D, aspartic acid; P, phosphate residue.

Understanding the molecular mechanisms of signal transduction pathways in the soil nitrogen-fixing microorganisms could contribute to better use of these bacteria in bioremediation and N_2_ fixation, which are important processes for the production of high-quality crops (rendering them independent from artificial N fertilization and increasing the amount of N available to plants). However, most studies currently focus on elucidation of the roles of TCSs in pathogenic bacteria (Gao, Mack & Stock, 2007). Sequence analysis of prokaryotic genomes demonstrated that TCSs exhibit unusual complexity and variability. These systems are composed of histidine kinases (HKs), which are sensor proteins located in the bacterial membrane, and RRs involved in the regulation of gene expression. The majority of HKs and RRs of individual TCSs in microorganisms are encoded by genes, which are located in the same operon and can be easily identified. However, over 15% of genes coding for RRs in bacteria occur individually (not grouped in operons) (Schaller, Shiu & Armitage, 2011). The architecture of TCS pathways in microorganisms can be extremely diverse. One of the simplest models is a TCS in which one HK capturing the signal of extracellular origin and one RR, which is the most common transcription factor (TF) that regulates the expression of a single target operon, are cognate (Fig. 1). Another model involves one HK activating one RR protein and affecting the expression of many different operons (even up to 30% of the bacterial genome, e.g., during bacterial entry into a latent state or a metabolism change from aerobic to anaerobic). Other TCS systems are also known, where one HK regulates many RRs or multiple HKs regulate only one RR (Schaller, Shiu & Armitage, 2011). Four main steps in the signal transduction cascade via TCSs can be distinguished: (I) signal detection, (II) sensor kinase activation, (III) phosphate group transfer to a regulatory protein, and (IV) response generation (Zschiedrich, Keidel & Szurmant, 2016) (Fig. 1).

HKs are homodimeric integral membrane proteins containing two transmembrane helices and with the N-terminus located in the cytosol. The sensor domain of these proteins is located between the transmembrane helices and shows a low degree of sequence similarity, in contrast to the other domains. The sensor domain may be located in the cytosol, cell membrane, or outside the cell, where it is involved in the recognition of extracellular signals and changes in the bacterial envelope (Wang, 2012). HAMP domain, which is commonly found also in other enzymes (named after the first letters of enzymes in which it occurs: His kinases, adenylate cyclases, methyl-accepting proteins, and phosphatases), is C-terminal to the second transmembrane helix. This domain connects the transmembrane helix 2 and His phosphorylation domain DHp (dimerization His phosphotransfer). It is the most conserved portion of the HK, which contains His, and is a signature motif of this type of enzyme. The DHp domain consists of two helices that form a hairpin-type structure, and a His residue that is also a site of HK autophosphorylation, located within the first helix. After ATP binding by the HK, the *γ*-phosphate group is transferred to histidine in the DHp domain. The catalytic domain possesses five conserved motifs, designated as N, G1, F, G2, and G3, which together with the H-frame are determinants for HK classification. The catalytic and ATP-binding domain (CA) is located at the C-terminus of the protein. The signature motifs within the HKs and phylogenetic analyses of HK protein sequences resulted in the identification of 11 major families of enzymes involved in TCSs. Further, recently, Karniol and Vierstra (Karniol & Vierstra, 2004) identified a new family of HKs, which share homology with protein BphP2 of *Agrobacterium tumefaciens*, whose phytochrome-sensing domain is involved in light perception. This type of HK lacks the F motif, although it does contain conserved amino acid residues in other motifs (e.g., histidine in the N motif and a tryptophan-X-glutamic acid motif in the G1 motif). Alterations of the above motifs were also detected in other members of *α*- and *β*-Proteobacteria, including bacteria from the *Rhizobiaceae* family (Karniol & Vierstra, 2004).

**Figure 2:**
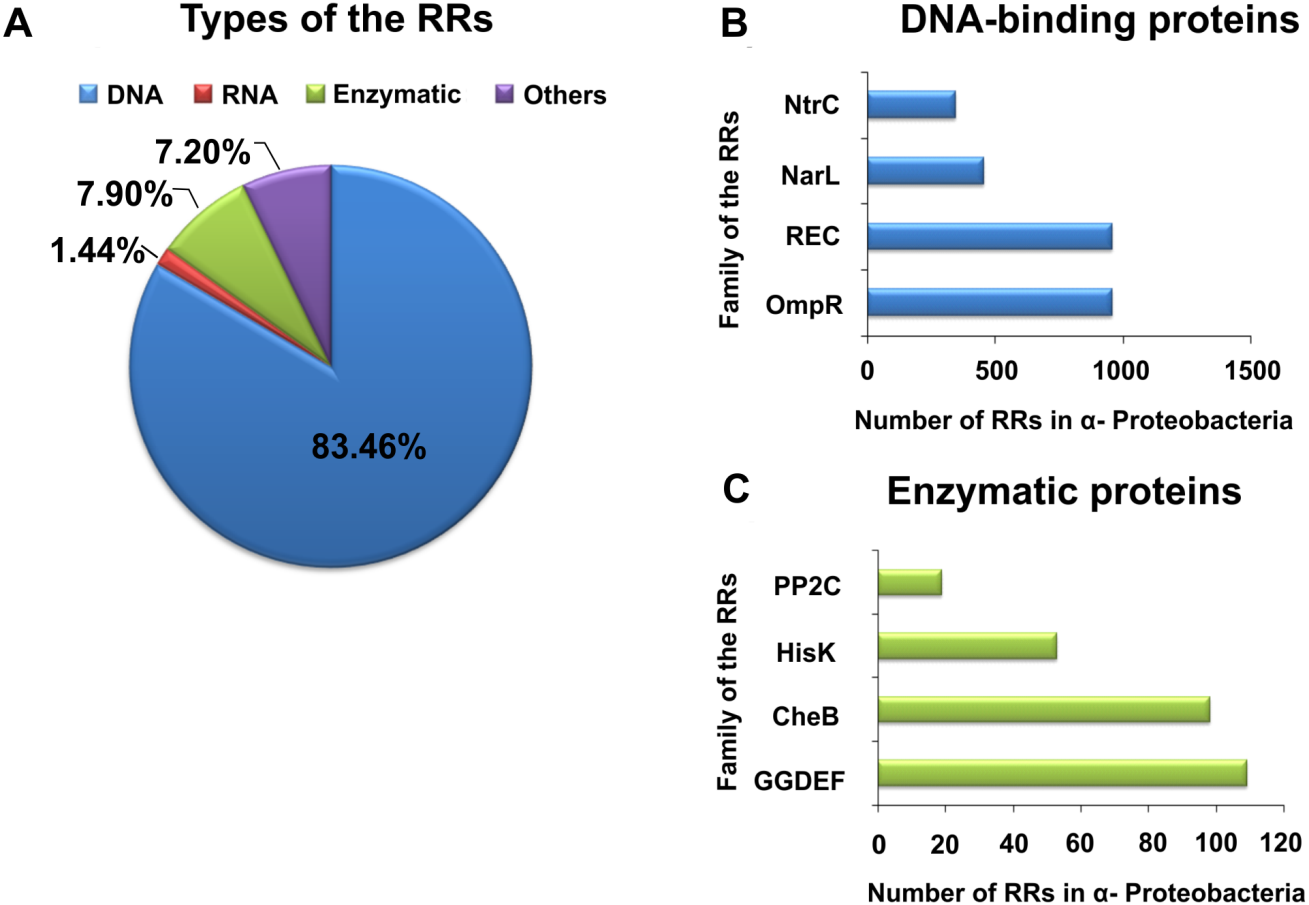
Global classification of bacterial RRs occurring in *α*-Proteobacteria in respect to their regulatory functions. (A) Numbers of individual-type RRs (DNA-binding, RNA-binding, enzymatic proteins, and of other cellular functions); (B) Numbers of chosen DNA-binding and (C) enzymatic RRs in *α*-Proteobacteria (prepared using data present in NCBI (2007)).

By contrast with HKs, RR proteins involved in the TCSs have only two domains, i.e., a receiver domain located at the N-terminus of the protein, which is responsible for binding the phosphate group, and an effector domain located at the C-terminus, which relays the signal in the transduction cascade. The receiver domain of RRs has a specific type of conservativeness in terms of its structure and sequence, while effector domains are characterized by high sequence variability, reflecting their different cellular functions. The vast majority of regulatory proteins of the TCS systems are TFs, which have different effector domains responsible for DNA, RNA, protein, and enzyme binding. In the receiver domain, an invariable Asp residue is present, which accepts the phosphate group coming from a cognate HK. Conformational changes in RRs induced by phosphorylation are next transferred to the effector domain, which consequently affects its activity (Gao, Mack & Stock, 2007; Mitrophanov & Groisman, 2008; Wang, 2012). Further, autophosphorylation regulates the duration of RR phosphorylated state, which translates into the half-life of the molecule, ranging from a few seconds to even an hour (Gao, Mack & Stock, 2007). Sequence analyses of bacterial genomes enabled structural and functional evaluation of the diversity of TCS RRs, which were grouped in a database and classified (the database contains nearly 9000 RRs) (NCBI, 2007). The largest number of endosymbiotic bacteria belong to the *α*-Proteobacteria; within this group, nearly 3530 RR proteins have been identified. Among them, a significant majority are TFs, which bind DNA (83.46%), and enzymes (7.90%) (Fig. 2).

**Table 1:** Role of TCSs in regulation of various cellular functions in representatives of rhizobial *α*-Proteobacteria (based on data from the KEGG and NCBI databases (https://www.genome.jp/kegg-bin/show_pathway?rlg02020*,*https://www.ncbi.nlm.nih.gov/; GenomeNet, 2019a; GenomeNet, 2019b)).

Family	Environmental factors	Sensor histidine kinases	Response regulators	Regulated proteins	Function	Bacteria	Reference
OmpR family	Phosphate limitation	PhoR	PhoB, PhoP	PhoA, PhoD	Phosphate assimilation	*Rhizobium* *Mesorhizobium* *Bradyrhizobium* *Sinorhizobium* *Agrobacterium*	Santos-Beneit (2015)
		SenX3	RegX3	PhoA, PstS		*Agrobacterium*	Xu et al. (2018)
	Mg^2+^ Starvation	PhoQ	PhoP	Unknown	Mg^2+^ assimilation	*Rhizobium* *Mesorhizobium* *Bradyrhizobium* *Sinorhizobium* *Agrobacterium*	Prost et al. (2007)
	Osmotic up-shift (K^+^)	EnvZ	OmpR	Unknown	Change in outer membrane (small and large holes)	*Rhizobium* *Mesorhizobium* *Bradyrhizobium* *Sinorhizobium* *Agrobacterium*	Yoshida, Cai & Inouye (2002), Yuan et al. (2011) and Sablok et al. (2017)
	Misfold protein	CpxA	CpxR	Unknown	Cell envelope protein folding and protein degradation	*Rhizobium* *Sinorhizobium*	Weatherspoon-Griffin et al. (2014)
	Copper ions	CusS	CusR	CusA	Copper efflux	*Rhizobium*	Gudipaty et al. (2012)
				CusC, CusF, CusB		*Mesorhizobium*	
				CusB, CusA		*Bradyrhizobium*	
				CusF, CusB, CusA		*Sinorhizobium*	
				CusF, CusB, CusA		*Agrobacterium*	
	Hormone like-molecules	QseC	QseB	MotA	Flagellum regulation	*Rhizobium* *Mesorhizobium* *Bradyrhizobium* *Sinorhizobium* *Agrobacterium*	Andres et al. (2013) and Corsini, Walker & Santini (2018)
	K^+^ limitation	KdpD	KdpE	KdpA, KdpB, KdpC	Potassium transport	*Rhizobium* *Mesorhizobium* *Bradyrhizobium* *Sinorhizobium* *Agrobacterium*	Prell et al. (2012) and Sablok et al. (2017)
	Catabolite repression	TctE	TctD	TctA, TctB, TctC	Tricarboxylates transport	*Rhizobium* *Mesorhizobium* *Sinorhizobium* *Agrobacterium*	Antoine et al. (2003)
		Unknown				*Bradyrhizobium*	
	Oxygen limitation	Unknown	ResD	CtaA	Respiration	*Rhizobium* *Sinorhizobium*	Svensson, Lübben & Hederstedt (1993)
		ResE	Unknown			*Bradyrhizobium*	
	Temperature	MtrB	MtrA	DnaA	DNA replication, Osmoprotection	*Rhizobium* *Mesorhizobium*	Wu et al. (2019)
	Acid condition	ChvG	ChvI	VirB, VirR,	regulation of acid-inducible genes and virulence	*Rhizobium* *Mesorhizobium* *Bradyrhizobium* *Agrobacterium*	Vanderlinde & Yost (2012)
				KatA, VirB, VirR		*Sinorhizobium*	
CitB family	Citrate	CitA	CitB	CitE, CitF, CitG	Citrate fermentation	*Rhizobium* *Mesorhizobium* *Bradyrhizobium* *Agrobacterium*	Scheu et al. (2012)
	C4- dicarboxyrate	DctB	Unknown	DctP	C4- dicarboxyrate transport	*Rhizobium* *Mesorhizobium* *Bradyrhizobium* *Sinorhizobium* *Agrobacterium*	Yurgel & Kahn (2004)
	Malate	MalK	Unknown	Unknown	Malate utilization	*Bradyrhizobium* *Mesorhizobium*	Kühnau et al. (1991)
			MalR			*Rhizobium* *Sinorhizobium* *Agrobacterium*	
LytTR family		LytS	LytR	LrgA, LrgB	Mureine hydrolase activity	*Rhizobium* *Mesorhizobium* *Sinorhizobium*	Behr et al. (2017)
		Unknown	LytR			*Bradyrhizobium Agrobacterium*	
NarL family	Nitrate/ Nitrite	NarX	NarL	NarG, NarH, NarI, NarJ	Nitrate reductase (Nitrogen metabolism)	*Rhizobium* *Bradyrhizobium*	Nohno et al. (1989)
		Unknown	NarP	FdnG, FdnH, FdnI	Formate dehydrogenase (Nitrogen metabolism)	*Mesorhizobium*	Nohno et al. (1989)
				FdnG, FdnI		*Bradyrhizobium* *Sinorhizobium*	
				Unknown		*Agrobacterium*	
	Salt stress	DegS	DegU	Unknown	Degradative enzymes	*Rhizobium* *Mesorhizobium* *Bradyrhizobium* *Agrobacterium*	Msadek et al. (1990)
NtrC family	Low nitrogen availability	GlnL	GlnG	GlnA	Nitrogen assimilation (glutamate metabolism)	*Rhizobium* *Mesorhizobium* *Bradyrhizobium* *Sinorhizobium* *Agrobacterium*	Shatters, Somerville & Kahn (1989)
		NtrY	NtrX	NifA	Nitrogen assimilation	*Rhizobium* *Mesorhizobium* *Bradyrhizobium* *Sinorhizobium*	Salazar et al. (2010)
				Unknown		*Agrobacterium*	
	C4- dicarboxyrate	DctB	DctD	DctA	C4- dicarboxyrate transport	*Rhizobium* *Mesorhizobium* *Bradyrhizobium* *Sinorhizobium* *Agrobacterium*	Golby et al. (1999)
Chemotaxis family	Attractant/ Repelent	MCP-CheA	CheY CheV CheB	Unknown	Flagellar motor switch adaptation	*Rhizobium* *Mesorhizobium* *Bradyrhizobium*	Jahreis et al. (2004)
			CheY CheB			*Sinorhizobium* *Agrobacterium*	
LuxR family	Oxygen limitation	FixL	FixJ	NifA, FixK	Respiration and nitrogen fixation	*Rhizobium* *Mesorhizobium* *Bradyrhizobium* *Sinorhizobium*	Monson, Ditta & Helinski (1995)
				FixK		*Agrobacterium*	
Others	Redox signal	RegB	RegA	PetA, PetB	Electron transfer system	*Rhizobium* *Mesorhizobium* *Bradyrhizobium* *Sinorhizobium* *Agrobacterium*	Bauer et al. (1998)
		RegS	RegR	NifA	Nitrogen assimilation	*Rhizobium* *Bradyrhizobium*	Lindemann et al. (2007)
				Unknown		*Agrobacterium*	

Galperin (2006) and Galperin (2010) showed that TFs belonging to the families OmpR, NarI, and NtrC account for almost 60% of all bacterial RRs. Similar, in *α*-Proteobacteria, these TF families constitute 51.64% of RRs. The regulatory domains of these RRs are very similar (from 20 to 30% sequence homology), and control the structure and function of various effector domains. These regulatory domains exist in two different conformations (active form, stabilized by phosphorylation, and inactive form), whose molecular surfaces differ in both states, allowing their various regulatory effects. In response to different environmental factors, symbiotic bacteria belonging to *α* Proteobacteria use various TCSs composed of a particular sensor HK and dedicated RR, which enables them to adapt to the changing habitat conditions. Examples of rhizobial TCSs and their function in various cellular processes are presented in Table 1. With respect to individual rhizobial species, genes encoding RRs from families REC, OmpR, NarL, NtrC, ActA, AmiR, GGDEF, HisK, and CheB constitute from 0.81 to 1.15% of the total number of genes in their genomes (e.g., *B. japonicum*, 1.15%; *Rhizobium etli*, 1.14%; *R. leguminosarum*, 0.98%; *S. meliloti*, 0.92%; *Rhizobium* sp. NGR234, 0.89%; and *M. loti*, 0.81%) (NCBI, 2007).

The availability of phosphate to bacteria in the soil varies, and ranges from 0.1 to 10 µM. Changes in the concentration of this element in the environment trigger activation of the TCS systems in soil bacteria and thus induce changes in the cell function. Phosphate limitation, in addition to other environmental factors, such as osmolality, ammonium availability, or the presence of flavonoids, affects the biosynthesis of extracellular surface polysaccharide (EPS) in symbiotic bacteria. The TCS system PhoR-PhoB is involved in the regulation of EPS synthesis in *Rhizobiaceace*. As indicated in *S. meliloti*, the PhoR protein is a HK that acts as a sensor of phosphate limitation in the environment, while the PhoB protein is a RR responsible for positive regulation of the expression of many genes associated with phosphate deficiency (among others, *wgaA*, *wggR*, and *wgeA)*. These genes are involved in the synthesis of EPS II (also called galactoglucan), which, as shown by numerous studies, is a signal molecule indispensable for biofilm formation, plant colonization, and establishment of effective symbiosis of *S. meliloti* with alfalfa (Bahlawane et al., 2008; Hagberg et al., 2016; Gao et al., 2012).

**Figure 3:**
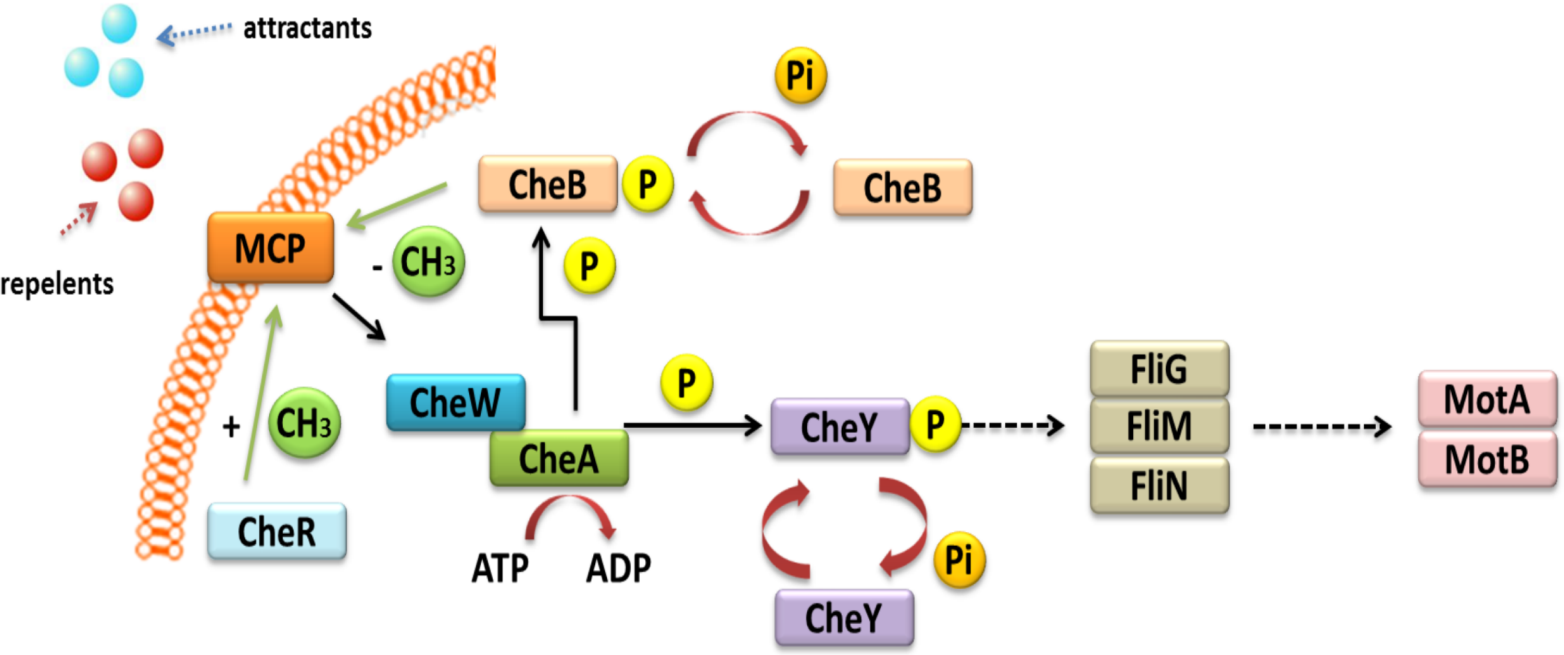
Mechanism of chemotaxis based on the TCS system in *α*-Proteobacteria (based on the KEGG database).

Other TCSs are also involved in the chemo-screening process in *α*-Proteobacteria. These TCSs are composed of transmembrane proteins, which are chemoreceptors, called methyl-accepting chemotaxis proteins (MCPs) (Fig. 3) (Meier, Muschler & Scharf, 2007; Zatakia et al., 2018). These proteins consist of two domains: a periplasmic domain that is a ligand and a signaling domain functioning as a cytoplasmic binding site, which is flanked by methylation regions. The cytoplasmic domain also contains a binding site for the HK CheA. This process is also mediated by adapter protein CheW. If a repellent compound is bound to the periplasmic domain, the conformation of the MCP changes; this relays the signal to the CheA protein and results in autophosphorylation. The phosphate group is then transferred to the CheY protein, which is a respiration regulator. By interacting with the FliM switch protein, it reverses the direction of the flagellum rotation, which changes the direction of bacterial migration in the environment (Alexander et al., 2010; Haslbeck, 2016). The phosphorylated CheY protein can spontaneously dephosphorylate, and its inactivation is accelerated by the CheZ protein, which has a dephosphatase activity. CheR and CheB are other Che proteins that are very important in bacterial chemotaxis. They are responsible for the methylation and demethylation of MCPs (Baker, Wolanin & Stock, 2006; Tambalo, Yost & Hynes, 2015). In the case of *E. coli* and *Salmonella,* five genes encoding MCP chemoreceptors have been identified in their genomes. They include genes for Tap protein, which traps dipeptides and pyrimidines; Tar, which reacts with maltose and aspartate; Tsr, which traps serine; Trg, which reacts with galactose and ribose; and Aer, which senses changes in the accessibility of oxygen. Four of these proteins are common to both these bacterial species (Tar, Tsr, Trg, and Aer) (Parkinson, Hazelbauer & Falke, 2015a).

In representatives of *α*-Proteobacteria, the majority of genes associated with motility, chemotaxis, and flagellum synthesis, as well as regulatory genes related with these processes, are located on the chromosome, usually in one or two operons. This type of organization provides coordinated expression of genes related to cell migration. In *R. leguminosarum*, *R. etli*, and *B. japonicum*, two complete operons (Che1 and Che2) encode proteins homologous to *E. coli* CheAWYRB (Aizawa, Harwood & Kadner, 2000; Miller et al., 2007). In the case of *S. meliloti*, only one of the Che operons is fully functional and encodes necessary chemotactic proteins. The second operon is located on plasmid pSymA and contains an incomplete set of genes (Meier, Muschler & Scharf, 2007). Only one complete Che operon is present in *A. tumefaciens*, while literature data indicate that *M. loti* harbors one but incomplete Che operon (Kaneko et al., 2000; Huang et al., 2018). Sequence analysis of the genomes of all these bacteria allowed determining the number of MCP chemoreceptors in different rhizobial species. The highest number of MCPs was identified in *B. japonicum* (36 MCPs), followed by bacteria belonging to the genus *Rhizobium* (28 MCPs), *A. tumefaciens* (20 MCPs), *Sinorhizobium* (9 MCPs), and only one chemoreceptor in *M. loti* (Tambalo, Yost & Hynes, 2015).

Other TCS systems are involved in atmospheric N_2_ fixation in rhizobia and are responsible for controlling the permeability of the bacterial envelope to oxygen in root nodules. One of the most common TCSs involved in these processes is FixL-FixJ, the activation of which occurs under micro-aerobic conditions, i.e., when the concentration of oxygen in the microbial cell is low (2%) (Monson, Ditta & Helinski, 1995; Bobik, Meilhoc & Batut, 2006). HK FixL has the ability to autophosphorylate using ATP as a phosphate donor. This is possible because of the presence of a heme group in the protein, to which the oxygen molecule binds and thus regulates the autophosphorylation. The phosphate group is then transferred to RR FixJ, which is a TF from the Crp-Fnr family. Phosphorylated FixJ controls a number of genes that influence signal transduction and adaptation of rhizobia to changing environmental conditions. According to literature data, FixJ controls nearly 74% of genes related to cell respiration under microaerobic conditions in *S. meliloti*. FixJ also acts as a positive regulator of the expression of the *fixK* gene, whose product is responsible for the regulation of the entire FixL-FixJ TCS system (Bobik, Meilhoc & Batut, 2006). Furthermore, FixK also controls the expression of the *fixT* gene, which encodes a protein that facilitates the dephosphorylation of FixL. The FixK protein itself controls over 90 genes related to cellular respiration and TCS regulation, as well as the response to stress factors, denitrification process, and cellular metabolism (e.g., transport of substances and Arg metabolism). Interestingly, FixK not only regulates the expression of genes involved in symbiotic processes but also affects the expression of genes related to the function of free-living bacteria (Bobik, Meilhoc & Batut, 2006; Reyes-González et al., 2016). The FixL-FixJ system also controls another transcription regulator involved in the expression of N_2_-fixing genes, the NifA protein. In bacterial cells, NifA controls a small number of genes (*fixABCX, nifHDKEX*, *nifB*, or *nifN*) whose expression is only detected in the bacteroid, but also genes involved in EPS production (*syrA* in *S. meliloti*) (Reyes-González et al., 2016). The occurrence of the FixL, FixJ, FixK, and NifA proteins is conserved among *α*-Proteobacteria; however, they differ with respect to the regulation mode and the set of the controlled target genes in different rhizobial species. However, some differences between various rhizobial species are observed, for example the expression of the *nifA* gene in *B. japonicum* is not dependent on the FixL-FixJ system, but depends on a completely different TCS system (RegS-RegR), which responds to redox potential changes in the cell (Bobik, Meilhoc & Batut, 2006; Stacey, 2007; Zamorano-Sánchez et al., 2012; Terpolilli, Hood & Poole, 2012).

## Phosphenolopyruvate-dependent Phosphotranspherase Systems (PTSs)

The PTS system in bacteria was first described over 50 years ago by Kundig, Ghosh & Roseman (1964), and is involved in the transport of carbohydrates in cell. This system is based on the transport of sugar components and other soluble substances from the environment to the bacterial cell, coupled with their simultaneous phosphorylation, which involves the participation of PEP (Kundig, Ghosh & Roseman, 1964; Saier, 2015). PTS systems are also involved in other physiological processes in bacteria, such as chemotaxis, regulation of carbon and nitrogen metabolism, and signal transduction (Kotrba, Inui & Yukawa, 2001; Pflüger-Grau & Görke, 2010; Saier, 2015).

**Figure 4:**
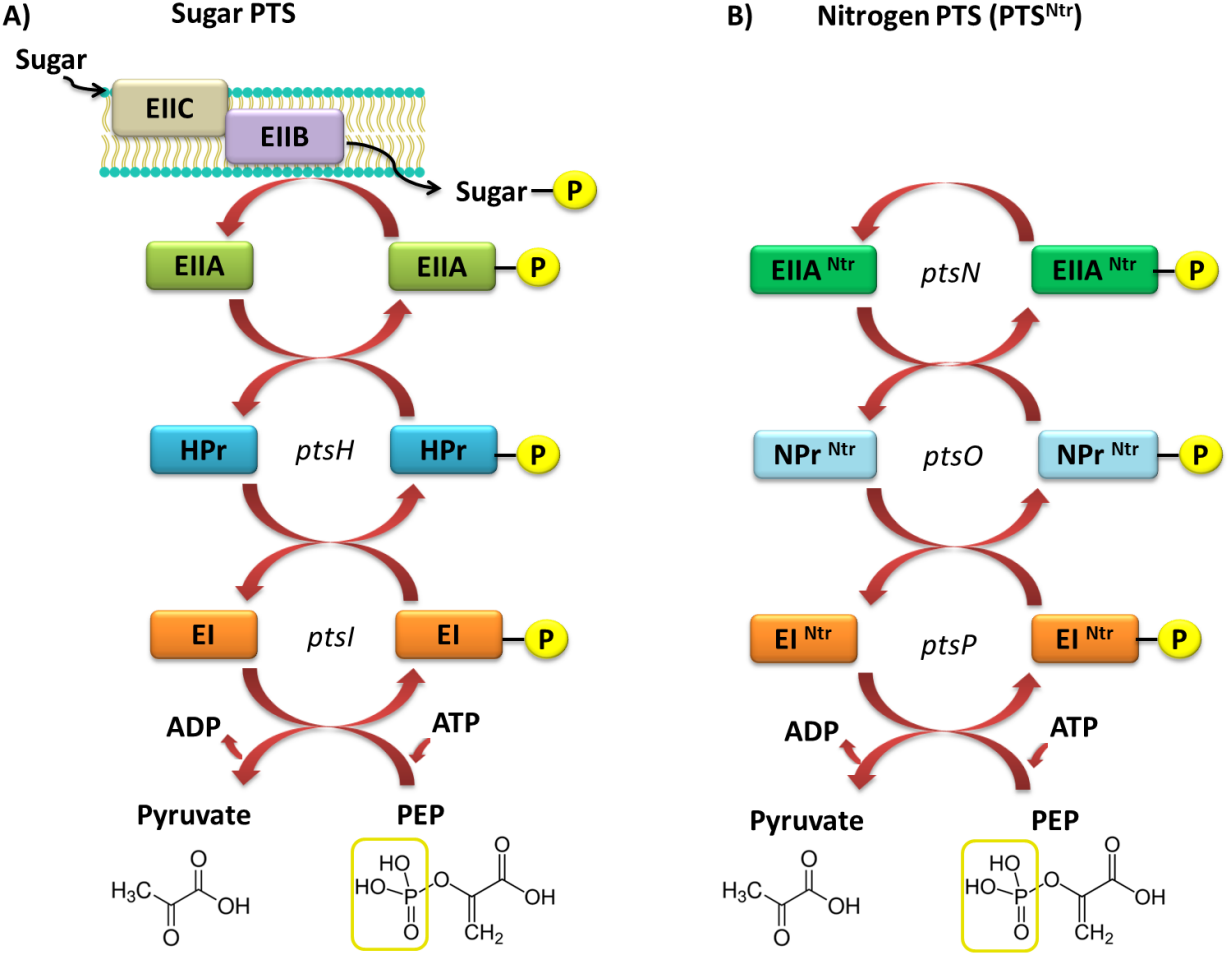
Models of the PTS systems in Gram-negative bacteria. (A) Classic PTS system responsible for the transport of carbohydrates into bacterial cells (example: *E. coli)*; (B) PTS system involved in regulatory functions in the bacterial cells (referred to as PTS^*Ntr*^) present in representatives of *α*-Proteobacteria (developed on the basis of *S. meliloti* species (Pflüger-Grau & Görke, 2010).

Currently, two types of PTSs are distinguished; the first one is associated with transport of most sugars and the second, PTS^Ntr^, is involved in regulatory processes in the cell. The classic PTS system is associated with transport of many different sugars, such as ketohexoses, aldohexoses, sugar alcohols, di- and trisaccharides, and aminosugars, into the cell. However, not all sugars can be transported through this system, for instance glycerol, glucuronide, and arabinose, which are transported by permeases and ATP-binding cassette (ABC) transporters. The PTS system is composed of four cytoplasmic proteins and one membrane protein. The first protein, called enzyme I (EI), is phosphorylated by PEP in the presence of Mg^2+^ ions on a His residue. Then, the phosphate group is transferred from EI to the HPr protein (to another conserved His residue). Both these proteins are involved in capturing the majority of PTS substrates and are subject to constitutive or partially induced expression in the cell (Deutscher, Francke & Postma, 2006; Deutscher et al., 2014; Choe et al., 2017). Another component of this system is the enzymatic complex II (EII), which consists of three to four protein domains called EIIA, EIIB, EIIC, and EIID. Phosphorylated HPr phosphorylates EII components. All these components have different substrate specificity with respect to carbohydrate transport and species specificity. Regulatory schemes within the PTS systems are not conserved among Gram-negative microorganisms, and are subject to numerous structural and functional modifications across species. Target proteins phosphorylated by PTSs are quite diverse and represent not only sugar transport systems or catalytic enzymes, but also TFs and HKs belonging to the TCSs. In most Gram-negative bacteria, the regulatory role of PTSs is reduced to the regulation of carbohydrate catabolism, which allows these microorganisms to adapt quickly to the changing and favorable carbon source, by phosphorylating PTS components. Based on sequence analyses of numerous genomes of bacteria belonging to the *α*-Proteobacteria, no fully functional PTS system, such as that in Gram-negative bacteria, has been found (Fig. 4).

In rhizobial representatives (*S. meliloti, M. loti, B. japonicum*, *R. leguminosarum* bv. *viciae*, and *S. fredii)*, only proteins homologous to the basic components of PTS were found, with the exception of EIIB and EIIC transport proteins (Li et al., 2016). Current literature data suggest that the incomplete PTS system in *α*-Proteobacteria is not involved in carbohydrate transport into the cell. Sugars and other soluble compounds are transported in these bacteria by widely distributed ABC-type transporters, which together with permeases, form up to 180 individual transport systems that are not yet fully characterized. The extremely complex network of the ABC-type systems in *α*-Proteobacteria reflects the requirements of these bacteria during their existence in the soil environment and symbiosis with legumes.

The second type of PTS (PTS^Ntr^) is present in all *α*-Proteobacteria representatives. This system contains the following components: EI^Ntr^ (PtsP), NPr (PtsO), and EIIA^Ntr^ (PstN) (Fig. 3) (Prell et al., 2012; Muriel-Millán et al., 2017). It has been intensively studied in recent years; nevertheless, the main functions of this system are still not fully understood. Based on the phenotypic effects of mutations present in genes encoding PTS^Ntr^ system components in the representatives of *α*-Proteobacteria, the system is involved in metabolic processes of rhizobia and their adaptation to stress conditions (Table 2).

**Table 2:** Effects of mutations in genes encoding PTS^Ntr^ system components in α-Proteobacteria representatives.

Bacterial strain	Mutated gene	Effects of the mutation	Reference
*S. meliloti* 1021	HPr (*ptsH*), *manX,* *ptsO*	Disorders in functioning of catabolic repression, inefficient symbiosis (nodules formed on alfalfa roots are inefficient in nitrogen fixation), decreased expression of *melA, agp* and *lac* operons (needed for the utilization of α- and β-galactosides), the enhanced production of high-molecular-weight succinoglycan	Pinedo, Bringhurst & Gage (2008)
*S. fredii*	*ptsP,* *ptsO*	Disorders in symbiosis (ineffective nodules formed on soybeans)	Li et al. (2016)
*S. fredii*	*ptsN*	No negative effects on the symbiosis with soybean	Li et al. (2016)
*R. etli*	*ptsN*	Decreased growth on carboxylic compounds, reduced production of melanin, and induction of *nifH* expression	Michiels et al. (1998)
*R. leguminosarum* bv. *viciae* 3841	*ptsP,* *ptsN,* *ptsO*	Change in the colony morphology from mucous to rough, reducing the range of ABC transporters	Prell et al. (2012), Untiet et al. (2013)
*B. japonicum* I110	*ptsP*	Reduction of oligopeptide uptake	King & O’Brian (2001)

**Figure 5:**
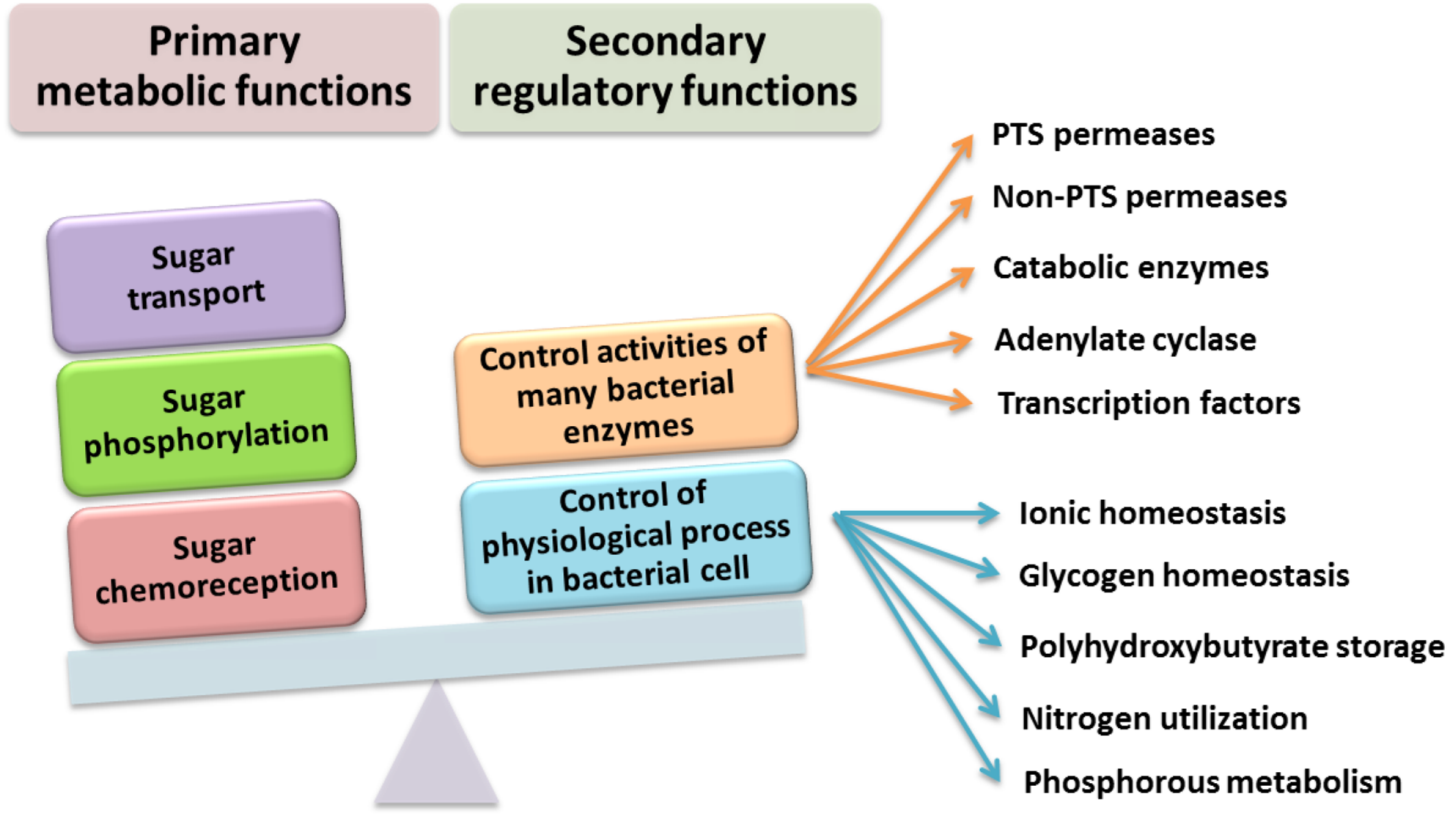
Functions performed by the PTS system in Gram-negative bacteria (based on data presented in Saier (2015).

It was experimentally determined that PTS^Ntr^ components also participate in the regulation of ABC-type transporters in *R. leguminosarum* bv. *viciae* 3841, and the presence of PtsP and PtsN proteins is required for their full activation. The PtsN protein also plays an important role in the activation of K^+^ ion transporters because it interacts (most likely in the non-phosphorylated state) with the KdpD sensor kinase. In addition to its involvement in the regulation of K^+^ ion concentration, PtsN regulates, directly or indirectly, many other cellular processes in various microorganisms, including rhizobia (Fig. 5). These processes include regulation of phosphate starvation (in *E. coli*), expression of nitrogen-fixing genes (*nif* genes in *Klebsiella pneumoniae)*, and accumulation of polyhydroxybutyrate (in *Azotobacter vinelandii)*. The current data also suggest that the presence of *α*-ketoglutarate glutamine can affect the phosphorylation status of the PTS^Ntr^ system by binding to the GAF domain (named after some proteins in which it is found: c**G**MP-specific phosphodiesterases, **A**denylyl cyclases, and **F**hlA) of the PtsP protein, as in *E. coli*. These observations suggest that PTS^Ntr^ can “sense” the availability of nitrogen.

## Phosphorylation of Tyr, Ser, and Thr Residues

Phosphoproteomic analyses performed for different bacterial species, including rhizobia, revealed that, in contrast with eukaryotes, bacteria contain several enzymes that are able to phosphorylate a wide range of amino acids (Mijakovic, Grangeasse & Turgay, 2016; Janczarek et al., 2018). As indicated for *S. meliloti* cells in the stationary phase, 96 unique phosphorylated sites in 77 proteins were identified; the ratio of detected phosphopeptides in these proteins was 63:28:5 Ser/Thr/Tyr (Liu, Tian & Chen, 2015). A similar ratio was also found in *E. coli* strain K12 (Ser 67.9%, Thr 23.5%, Tyr 8.6%), and is similar to those in human proteins (Ser 86.4%, Thr 11.8%, and Tyr 1.8%). The phosphorylation of these amino acids is dynamic and reversible, due to the action of appropriate phosphatases, which are thus involved in signal transduction pathways in the cell (Macek et al., 2008; Shi, 2009).

The process of phosphorylation and dephosphorylation of Tyr in bacteria is conducted by protein tyrosine kinases (TKs) and two types of tyrosine phosphatases (TPs). The former are conventional eukaryotic-type phosphatases, while the latter are acid phosphatases characterized by low molecular weight (LMW-TPs) (Macek et al., 2008). Genes encoding relevant pairs of TKs and TPs most often located in large operons, which are responsible for regulating the synthesis of surface polysaccharides [such as EPS and capsular polysaccharides (CPS)] or biofilm formation. These processes are involved in both the virulence of pathogenic bacteria and symbiotic interactions (as in the case of rhizobia) (Janczarek, 2011; Medeot et al., 2016; Marczak et al., 2017). Bacterial TKs are characterized as polysaccharide co-polymerases participating in the polymerization of EPS subunits or recurring O-antigen subunits in LPS molecules (Marczak et al., 2017). This group includes the following proteins: Wzc of *E. coli*, CpsD of *Streptococcus pneumoniae*, Ptk of *Acinetobacter johnsonii,* and AmsA of *Erwinia amylovora* (Soulat et al., 2007; Grangeasse, Nessler & Mijakovic, 2012; Nourikyan et al., 2015). Unfortunately, only a small number of TKs (e.g., ExoP of *S. meliloti* and PssP of *R. leguminosarum*) and TPs have been described in symbiotic bacteria (Mazur et al., 2002; Medeot et al., 2016). Additionally, there is no knowledge of the mechanisms and role of these proteins in the signal transduction pathway in rhizobia as well as target proteins that they regulate. The structure of TKs in symbiotic bacteria does not greatly differ from that of classical TKs, which are present in the majority of pathogenic bacteria. The catalytic domain is characterized by the presence of the Walker A and B motifs, and the absence of motifs characteristic of typical eukaryotic kinases. These enzymes also have the potential to autophosphorylate, which occurs in the Tyr-rich region located in the C-terminal part of these proteins (Grangeasse, Nessler & Mijakovic, 2012). As in the case of other kinases described herein, the donor of the phosphate group is ATP, and the degree of phosphorylation of bacterial TK determines its ability to interact with other proteins in the bacterial cell (Whitmore & Lamont, 2012). Among rhizobial TKs, the most detailed description has been provided for ExoP in *S. meliloti*, which is involved in the polymerization of EPS I (succinoglycan). This protein is encoded by the *exoP* gene, which is located in a large gene cluster (30 kb) on pSymB plasmid responsible for EPS I biosynthesis (this cluster includes 21 *exo* and *exs* genes) (Niemeyer & Becker, 2001). ExoP is a membrane protein consisting of an N-terminal periplasmic domain located between two transmembrane regions, and an additional cytoplasmic domain located at the C-terminus and containing ATP-binding motifs (Walker A and B motifs) (Niemeyer & Becker, 2001; Schmid, Sieber & Rehm, 2015; Medeot et al., 2016).

**Table 3:** Comparison of the biochemical and structural properties of ExoP proteins in selected rhizobial species.

Name of protein	Bacteria	Molecular weight (kDa)	Length (aa)	Sequence identity (%)/ Sequence similarity (%)	Hydrophobic amino acids (%)	Hydrophilic amino acids (%)	pI	Secondary structure of the protein	
								Number of *α*-helixes	Number of *β*-sheets
ExoP	*S. meliloti*	86.14	786	40/57	53.32%	46.68%	7.0	26	15
ExoP	*B. japonicum*	81.65	756	28/46	53.18%	46.30%	6.1	24	13
ExoP	*M. loti*	80.58	759	24/45	57.18%	42.82%	4.8	22	14
PssP	*R. leguminosarum* bv. *trifolii*	84.05	758	99/99	52.11%	47.89%	5.2	25	13
ExoP	*A. tumefaciens*	85.34	782	40/59	52.43%	47.57%	5.9	26	15

**Figure 6:**
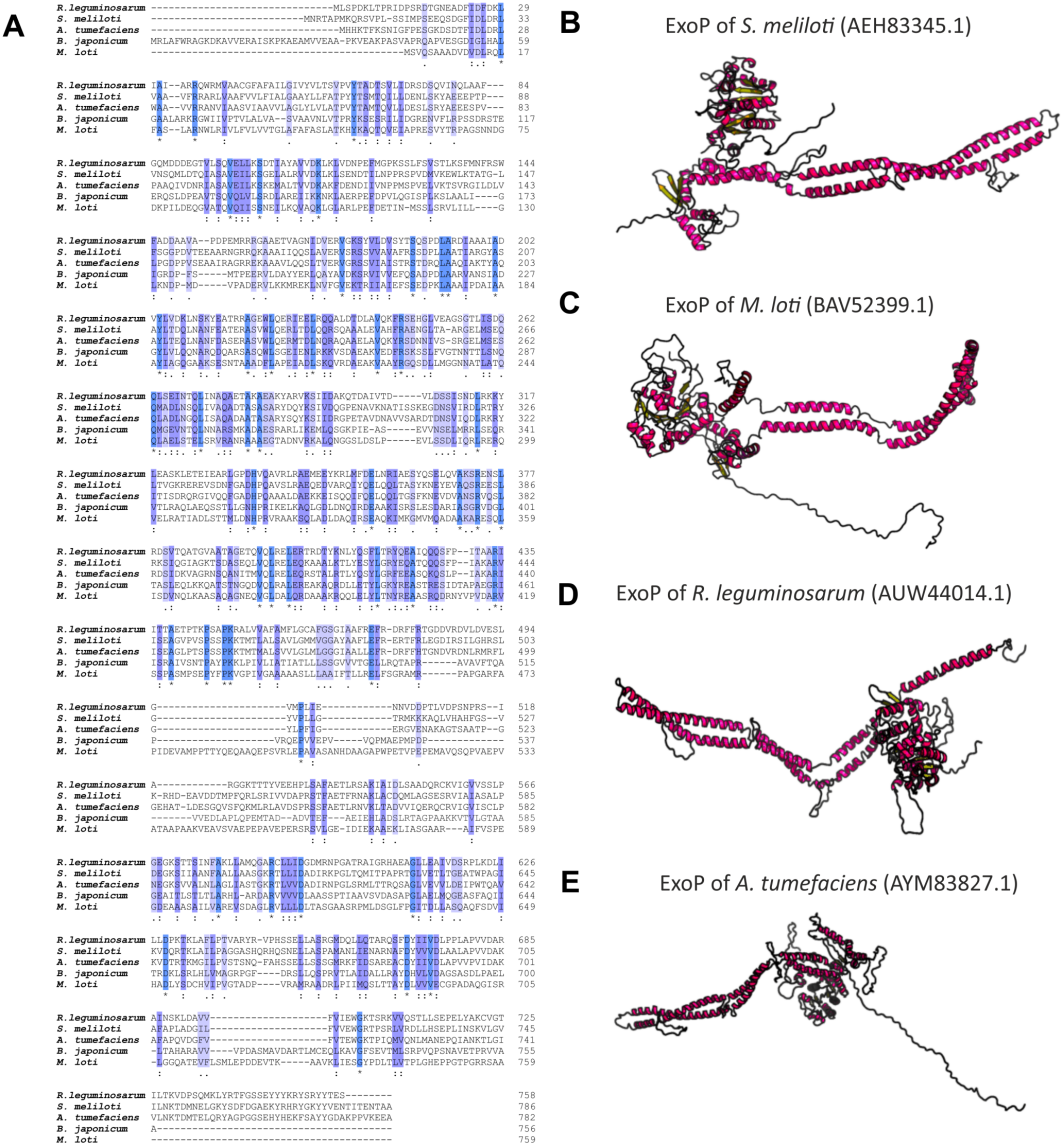
Characteristics of ExoP proteins in selected rhizobial species. (A) Alignment of sequences of ExoP proteins from *S. meliloti, B. japonicum, M. loti, R. leguminosarum*, and *A. tumefaciens*, developed in the ClustalOmega program; (B–E) theoretical models of ExoP proteins from selected symbiotic bacteria generated by the RaptorX program.

Numerous studies have shown that a mutation in the *exoP* gene in *S. meliloti* blocks the polymerization of EPS I subunits and results in a significant reduction in the production of this polysaccharide (increased LMW fraction of EPS I in relation to the HMW form). This was caused by the fact that only the N-terminal part of the protein is expressed in the *exoP* mutant. Despite the changes in the ratio between HMW and LMW EPS I produced, as well as the adhesion ability of the *exoP* mutant, this bacterium can infect the host plant. Homologs of *S. meliloti* ExoP are also present in other representatives of *α*-Proteobacteria, including *B. japonicum* (Becker & Pühler, 1998), *M. loti* (Kaneko et al., 2000), *Rhizobium* spp. (Staehelin et al., 2006), or *A. tumefaciens*, where these proteins serve similar functions as that in *S. meliloti.* In spite of their functional similarity in different bacteria, ExoP proteins in these microorganisms differ in biochemical properties and sequence (i.e., molecular mass, length, amino acid composition, and isoelectric point) (Table 3). Analysis of the amino acid sequences of ExoP proteins from rhizobial strains described to date indicates a diverse degree of sequence similarity (from 24% to 99% identity and from 45% to 99% sequence similarity, depending on the species) (Table 3, Figs. 6A and 6B). These differences are also highlighted by the analysis of the secondary structure of these proteins, revealing a variable number of *α*-helices and *β*-sheets in these proteins (Table 3) (based on NetSurfP-2.0, 2007 analysis), and are evident during generation of theoretical three-dimensional protein models (Fig. 6) (based on the RaptorX, 2011 analysis).

The EPS copolymerase in *R. leguminosarum* is PssP (PCP2a), which is similar to the *S. meliloti* ExoP protein. The *pssP* gene encoding TK is located in the terminal part of the Pss-I region, in which genes responsible for the synthesis, polymerization, and export of EPS are grouped (Mazur et al., 2002). PssP is 746-aa long protein (molecular mass of 82.39 kDa) located in the bacterial internal membrane. The structure of PssP is highly similar to that of ExoP proteins. In its structure, a hydrophilic N-terminal domain surrounded by two transmembrane regions and a C-terminal domain containing Walker A and B motifs have been identified. The only major difference is that the PssP protein does not contain a Tyr-rich region present in ExoP (Ivashina & Ksenzenko, 2012). The mutation in the *pssP* gene, similar to the mutation in the *exoP* of *S. meliloti*, affects the amount of EPS produced by *R. leguminosarum*. A deletion of the entire *pssP* gene in *R. leguminosarum* strain TA1 results in a complete inhibition of EPS production, and alteration of colony morphology (rough, non-fluid colonies are formed) in comparison with the wild type (mucoid colonies). It also affects the ability of the mutant to interact with the host plant (no colonization of clover roots) and fix atmospheric N_2_. Further, mutant strains synthesizing PssP that lacks the C-terminal region produce a reduced amount of EPS, with a changed ratio of the HMW fraction to the LMW fraction of this polymer (dominance of the LMW EPS fraction) (Mazur et al., 2002; Marczak et al., 2017).

Currently, very little information is available for the TPs in *Rhizobiaceae*. To date, the literature contains one example gene, the chromosomal gene *SMc02309* in *S. meliloti*, which encodes a potential TP. Bioinformatics analyses have shown that a protein encoded by this gene shares a high (43%) sequence identity with the *E. coli* Wzb protein. Biochemical analyses have confirmed that SMc02309 can hydrolyze an artificial substrate p-NPP, used in *in vitro* assays, allowing determination of its phosphatase properties. Furthermore, studies with *S. meliloti* SMc02309 have shown that this protein is able to dephosphorylate ExoN (UDP-glycosyl pyrophosphorylase involved in UDP-glucose synthesis) on Tyr residues (Medeot et al., 2016).

Another important issue in bacterial regulatory pathways is phosphorylation of Ser and Thr. Although Ser and Thr are among the most frequently phosphorylated amino acids in bacteria, the knowledge of kinases and phosphatases responsible for their phosphorylation/dephosphorylation is insufficient. In bacteria, two types of Ser kinases can be distinguished: Hanks-type (commonly referred to as eukaryotic-like Ser/Thr) kinases (STKs) and atypical Ser kinases. The first type encompasses cytoplasmic and membrane proteins whose location is variable, and depends on the enzyme structure and occurrence of additional sub-domains that also affect its activity. This group of enzymes contains 12 specific motifs that were defined by the discoverer of the Hanks proteins. As in the case of classical kinases, the catalytic domain participates in the binding of the phosphate group from the donor molecule (ATP) in the N-terminal part of protein, while the C-terminal part is responsible for the interaction of the protein with substrate molecules and is involved in the transfer of this group. Thus far, a large number of STKs (approximately 60) has been described. They mainly originate from Gram-positive and Gram-negative bacteria pathogenic to human, which emphasizes the importance of phosphorylation in all microorganisms and how much research is still needed to fully understand this regulatory process. The functional range of proteins regulated by STKs is extremely broad and is related to many diverse cellular processes, such as cell division, central metabolism control, envelope biogenesis, regulation of ABC transport systems, regulation of translation and transcription, and stress responses (e.g., heat shock, sporulation, osmotic stress, etc.) (Janczarek et al., 2018).

Unfortunately, the knowledge of STKs and Ser/Thr phosphatases (STPs) in symbiotic bacteria is still insufficient. At present, only one rhizobial STK has been described in the literature (i.e., PrkA STK in *Mesorhizobium alhagi*, a bacterium able to infect *Alhagi sparsifolia* naturally occurring in the Mediterranean area) (Chen et al., 2010; Liu et al., 2016). PrkA is an extremely conservative STK with homology to STKs not only in *E. coli*, but also in *Bacillus subtilis* and *Mycobacterium tuberculosis*. This protein plays an important role in the regulation of bacterial metabolism under stress conditions (such as osmotic or acid stress). PrkA of *M. alhagi* consists of 649 amino acids (molecular mass of 74.73 kDa) and is involved in the adaptation of rhizobial cell to stress conditions caused by increased salinity in the environment (Liu et al., 2016).

**Table 4:** Comparison of the biochemical and structural properties of PssZ proteins in selected rhizobial species.

Protein	Bacteria	Molecular weight (kDa)	Length (aa)	Sequence identity (%)/ Sequence similarity (%)	Hydrophobic amino acids (%)	Hydrophilic amino acids (%)	pI	Secondary structure of the protein	
								Number of *α*-helixes	Number of *β*-sheets
PssZ WP_026230739	*R. leguminosarum* bv. *trifolii* Rt24.2	29.28	263	-/-	58.55	41.45	8.6	11	10
ABC92003	*R. etli* CFN 42	28.95	260	92/94	56.92	43.08	8.2	13	12
WP_085738086	*Rhizobium sp.* CIAT894	29.26	263	94/96	57.41	42.59	8.4	12	12
WP_063898332	*M. loti*	28.87	256	47/60	54.68	45.32	6.8	10	11
EKJ95978	*B. lupini* HPC(L)	29.4	263	46/62	57.41	42.59	6.9	13	10
WP_012652475	*A . tumefaciens*	28.92	263	50/66	55.51	44.49	5.4	11	12

**Figure 7:**
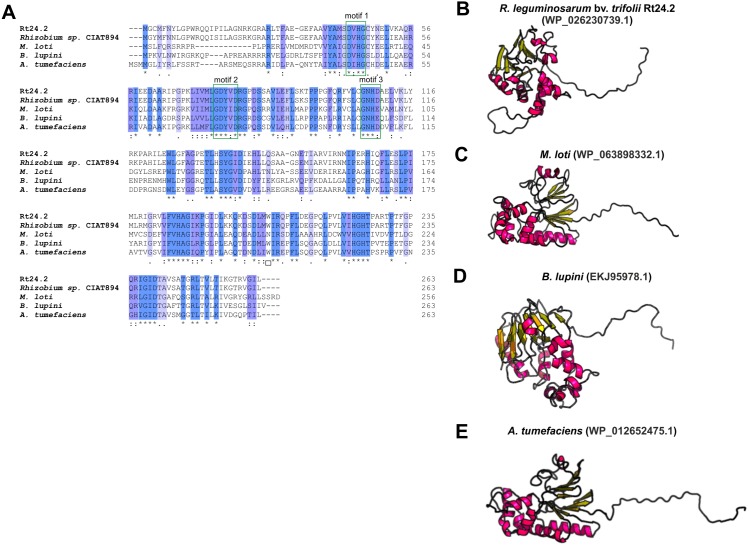
Characteristics of the PssZ proteins in selected rhizobial species. (A) Alignment of sequences of PssZ proteins from *B. lupini, M. loti, R. leguminosarum*, ** and *A. tumefaciens* developed in the ClustalOmega program; (B–E) theoretical models of PssZ proteins from selected symbiotic bacteria generated by the RaptorX program.

Even less information can be found for rhizobial STPs. The currently available literature data pertain to a small percentage of these enzymes, mainly focusing on human pathogenic bacteria (in particular, Gram-positive bacteria; only 30% of STPs described in the literature originate from Gram-negative bacteria). Bacterial STPs are classified into two phosphatase families: classical bacterial phosphoprotein phosphatases (PPPs) or protein metallophosphatases (PPMs). These enzymes play a variety of functions in the bacterial cell, as do the corresponding STKs. STPs are involved in the following processes: cell growth and division, cell signaling, sporulation, biofilm formation, motility, and regulation of transcription and translation. Interestingly, the first and only STP described to date in a symbiotic bacterium from the *Rhizobiaceae* family is PssZ from *R. leguminosarum* bv. *trifolii*. This protein is encoded by the *pssZ* gene located in the Pss-I region, which is responsible for the synthesis and export of EPS. PssZ is 263-amino acid-long protein with a molecular mass of 29.28 kDa (isoelectric point of 8.62) (Table 4). The protein shows a various degree of homology with proteins from other symbiotic bacteria (46–90% for bacterial strains from the genus *Rhizobium*; 44–46% for the genus *Sinorhizobium*; 47–54% for the genus *Agrobacterium*; and 44–47% for the genera *Bradyrhizobium* and *Mesorhizobium)*. Bioinformatics analyses of the secondary structure of PssZ have shown that this protein contains 11 *α*-helixes and 10 *β*-sheets, as well as three motifs characteristic for and conserved in PPMs (-GDXHG-, -GDXVDRG-, and -GNHE-) (Fig. 7). Furthermore, amino acids characteristic for STPs from the PPM family (His at positions: 45, 108,186, and 225; and Asn107, Asp43, and Asp76), which are responsible for the binding of metal ions magnesium and manganese have also been identified. To determine the role of the PssZ protein in the functioning of rhizobial cells, a spontaneous mutant harboring a Tn*5* transposon insertion in the *pssZ* gene was evaluated. Comparative transcriptomic analyses of the *pssZ* mutant and the wild-type strain showed that the lack of functional PssZ protein affects multiple cellular processes, including transcription, translation, and signal transduction, as well as cell division and motility. Furthermore, the mutation in *pssZ* led to the inhibition of the synthesis of surface polysaccharides, such as EPS and CPS, and substantially reduced the amount of produced gel-forming and neutral polysaccharides. The lack of functional PssZ also significantly affects the growth of *R. leguminosarum*, prolonging the generation time, and negatively influences cell motility. The lack of this protein also dramatically affects the effectiveness of symbiosis of this bacterium with its host plant, i.e., red clover (*Trifolium pratense*) (the *pssZ* mutant induces the formation of deformed root nodules, which are unable to reduce atmospheric N_2_) (Lipa et al., 2018; Lipa, Vinardell & Janczarek, 2019).

## Conclusions

Phosphorylation is a key mechanism that enables microorganisms to exist in various ecological niches, and to sense and respond to changing environmental conditions. Sensing and transduction of different signals (both external and internal) allow bacteria to adapt to different environments. These processes are conducted by various regulatory systems, including TCSs, PTSs, and STKs/STPs. Several studies indicate that regulatory pathways controlled by phosphorylation/dephosphorylation processes play an essential role in the regulation of various cellular processes in symbiotic bacteria, such as growth and cell division, cell wall biosynthesis, biofilm formation, stress response, metabolic processes, symbiotic interaction with legumes, and nitrogen fixation. Reversible phosphorylation of many protein targets involved in bacterial signaling and physiology is catalyzed by enzymes belonging to different families of kinases and phosphatases. However, the current knowledge of rhizobial enzymes involved in the phosphorylation/dephosphorylation processes, environmental signals that trigger the signaling cascade, and the mechanisms that regulate the crosstalk between these enzymes is insufficient. Studies in this research field will provide understanding of the function of prokaryotic regulatory networks and their role in adaptation of rhizobia to different ecological niches, such as the soil, rhizosphere, and legume root nodules. Thus, the most important aspects of the role of these regulatory systems that should be addressed are understanding their molecular mechanisms of action, as well as the relationships between them. These data will allow us to understand the complexity of rhizobial sensing and response to various environmental stressors that may ensure better application of these bacteria in sustainable agriculture in the future.

## Additional Information and Declarations

### Data Availability

The following information was supplied regarding data availability: This is a literature review and does not have raw data.
